# Ascites regression following neoadjuvant chemotherapy in prediction of treatment outcome among stage IIIc to IV high-grade serous ovarian cancer

**DOI:** 10.1186/s13048-016-0294-z

**Published:** 2016-12-02

**Authors:** Xia Xu, Fei Deng, Mengmeng Lv, Binhui Ren, Wenwen Guo, Xiaoxiang Chen

**Affiliations:** 1 Department of Chemotherapy, Jiangsu Cancer Hospital, Nanjing, Jiangsu 210009 People’s Republic of China; 2 Department of Gynecologic Oncology, Jiangsu Cancer Hospital, 42# Baiziting street, Nanjing, Jiangsu 210009 People’s Republic of China; 3 Department of Thoracic Oncology, Jiangsu Cancer Hospital, Nanjing, Jiangsu 210009 People’s Republic of China; 4 The Second Affiliated Hospital of Nanjing Medical University, Nanjing, Jiangsu 210009 People’s Republic of China

**Keywords:** High grade serous ovarian cancer, CA-125, Ascites, Primary debulking surgery, Interval debulking surgery

## Abstract

**Background:**

No consensus exists on the outcome-related factors of interval debulking surgery (IDS) in patients with advanced high-grade serous ovarian cancer (HG-SOC) who underwent neoadjuvant chemotherapy (NAC). This study aimed to explore the optimal timing for IDS and the prognosis-associated factors of International Federation of Gynecology and Obstetrics stage IIIc to IV HG-SOC patients.

**Methods:**

A total of 160 IIIc to IV stage HG-SOC patients were retrospectively analyzed. Patients with large volume ascites underwent NAC and subsequent IDS from the Jiangsu Institute of Cancer Research between 1993 and 2013. The outcome of IDS-associated factors was explored by logistic regression. To predict IDS outcome, the potential values of serum CA-125 levels and CA-125 decreasing kinetics were determined by the receiver operating characteristic curve. The associations between survival durations and covariates were assessed by Cox proportional hazards model and log-rank test.

**Results:**

Optimal IDS was achieved in 80.6% of HG-SOC patients who underwent NAC. Multivariate analyses revealed that ascites regression (*p* = 0.01), serum CA-125 level (*p* = 0.02), and CA-125 decreasing kinetics (*p* = 0.01) were independent optimal IDS predictors. CA-125 decreasing kinetics, IDS outcome, and ascites volume were independently associated with overall survival (OS) (*p* = 0.04, *p* < 0.01, *p* = 0.03, respectively) and progression-free survival (PFS) (*p* < 0.01, *p* < 0.01, *p* = 0.02, respectively). Patients who exhibited disappearance of ascites (<500 ml) had longer PFS (19.7 months vs.14.9 months) and OS (32.1 months vs. 26.0 months) than patients who exhibited residual ascites (≥500 ml). Subsets with higher CA-125 decreasing kinetics (≥2.2) had longer PFS (21.4 months vs.13.1 months) and OS (29.6 months vs.26.8 months) than counterparts (kinetics < 2.2).

**Conclusions:**

Ascites regression and CA-125 decreasing kinetics were independently associated with surgical outcome and prognosis in advanced HG-SOC patients who underwent NAC.

## Background

Epithelial ovarian cancer (EOC) is the most lethal gynecological cancer in North America, Western Europe, and China [1, 2]; its mortality rate has insignificantly decreased since the 1970s [3]. A total of 41,516 EOC cases were registered in China in 2011[4]. In 2015, EOC resulted in an estimated 21,290 cases and 14,180 deaths in the United States [5].

EOC is a heterogeneous tumor group, with high-grade serous ovarian cancer (HG-SOC) as the archetype and main cause of the high fatality rate [6]. The treatment outcome of HG-SOC is very poor because this disease is most commonly diagnosed in advanced stages at initial treatment. The current standard therapeutic strategy for advanced ovarian cancer is maximum primary debulking surgery (PDS) followed by frontline taxane plus carboplatin chemotherapy. Cytoreduction aims to achieve optimal debulking, as the amount of residual tumor after this procedure is one of the most important prognostic factors for the survival of women with HG-SOC. Over the past 30 years, the definition of optimal cytoreduction has changed from residual tumors <1–2 cm to absent macroscopic disease [7–11].

An optimal surgical procedure is not always possible for patients with advanced-stage HG-SOC (IIIc to IV) with poor performance status or medical contraindications. Morever, extensive internal organ resection and major blood loss are associated with a high risk of morbidity. To reduce the patient’s tumor burden and improve therapeutic effect by allowing further surgery, surgeons investigated the possibility of reducing tumor size with two to four cycles of neoadjuvant chemotherapy (NAC). No consensus exists on the optimal timing indicators for interval cytoreduction surgery (IDS) in advanced ovarian cancer, including tumor markers, ascites volume, imaging information, and patients’ status [12, 13].

Women who have a high perioperative risk profile or a low likelihood of achieving cytoreduction to <1 cm (ideally to no visible disease) were suggested to receive NAC. Chemotherapy may increase the ratio of patients suitable for IDS; the rates of optimal resection in IDS after induction chemotherapy had been reported to range from 77 to 94% [14–19]. Patients who underwent IDS after NAC had lower morbidity, lower requirement of intensive care unit admission, lower duration of hospital stay, and higher quality of life than the patients who underwent PDS.

A consensus exists that a complete resection of all macroscopic disease (at PDS or IDS) is an independent predictor of progression-free survival (PFS) and overall survival (OS). Unlike the advantages for resectability and response rates demonstrated by most studies, there is no evidence of survival advantage demonstrating that NAC followed by IDS is inferior to the gold standard procedure, and the role of NAC has been debated for years [20, 21]. Different EOC subtypes have distinct clinical characteristics and prognosis [6, 22–25]. Furthermore, different research methods utilized distinct recruitment standards that varied from one to another in terms of ascites volume, gross tumor burden, and the International Federation of Gynecology and Obstetrics (FIGO) stage. No published comparative study currently exists on these issues in HG-SOC. Therefore, evaluating the value of platinum-based NAC in a large HG-SOC sub-population is urgently needed.

In the present study, we retrospectively analyzed clinicopathological factors in patients with HG-SOC who received NAC/IDS at the Jiangsu Institute of Cancer Research (JICR, PRC).

## Methods

### Study population

A retrospective chart review was conducted to identify all patients diagnosed with HG-SOC treated at the JICR from January 1, 1993 to December 31, 2013. The analysis included all the patients treated at the institution during that period and who met the recruitment standards.

Multidisciplinary team (MDT) consists of two gynecologic oncologists, two pathologists, one radiologist and one medical oncologist was set to indentify women with a high perioperative risk profile or a low likelihood of achieving optimal cytoreduction should receive NAC. Before NAC is delivered, 76 patients have histologic confirmation of an invasive ovarian, fallopian tube, or peritoneal cancer from core biopsy. In exceptional cases, when a biopsy cannot be performed, cytologic evaluation combined with a serum CA-125 to carcinoembryonic antigen (CEA) ratio >25 is acceptable to confirm the primary diagnosis in 62 cases. In other 22 cases with CA-125 to CEA ratio was 25 or lower, patients had to have an additional barium enema or colonoscopy, gastroscopy or radiologic examination of the stomach, and mammography to rule out a potential metastatic primary malignancy. Under these criteria, the incidence of other malignancies confirmed by IDS was 5%; and only 8 patients were excluded in further analysis.

The recruit criteria of the present study were as follows: patients must be histological confirmed stage IIIc to IV ovarian, fallopian tube, or peritoneal high-grade serous cancer by IDS; An additional inclusion criterion of large volume ascites estimated by ultrasound and confirmed by surgical procedure was set to observe the regression of ascites by NAC (35 frailty cases underwent slow-release evacuation procedure before NAC treatment for the intolerable abdominal distension were excluded); patients who underwent PDS were excluded; patients who underwent non-platinum NAC were excluded; patients who did not undergo entire primary therapy procedure were excluded; patients with preoperative serum CA-125 levels < = 35 U/ml were excluded; laparoscopic proved HG-SOCs were excluded. The number of NAC cycles was administered based on the MDT’s decision. During 1993–1997, cisplatin (50 mg/m^2^), farmorubicin (50 mg/m^2^), and a cyclophosphamide (500 mg/m^2^) combined regime was administrated to 18 HG-SOCs every 3 weeks. After 1997, 142 cases underwent carboplatinum (area under the curve 5–6) and paclitaxel (135–175 mg/m^2^) regime every 3 weeks. An IDS was scheduled approximately 2 to 4 weeks after NAC. Patients were then treated with at least three to four additional chemotherapy cycles with the same regimen as NAC. Disease progression was evaluated by computed tomography (CT, all cases)/magnetic resonance imaging (MRI, 49 cases) or positron emission tomography imaging (PET/CT, 25 cases) of the abdomen and pelvis before initiating of frontline or second-line chemotherapy. The patients’ follow-up plan after the completion of primary treatment included clinical assessment and serum marker measurement, as mentioned previously [22–24].

### Clinicopathological characteristics

The clinicopathological data of the patients were reviewed and the following data were collected: age; tumor grade; histology; tumor stage; serum CA-125 and CEA levels during diagnosis, therapy, and follow-up; NAC regime, courses, and clinical or pathological responses; optimality of cytoreductive surgery; and disease status at the last follow-up. Ascites volume was estimated by ultrasound and confirmed by surgical procedure. Regression was defined by as an ascites volume < 500 ml. CA-125 decreasing kinetics was defined as the ratio of the initial serum CA-125 level divided by the preoperative serum CA-125 level. Surgical staging followed the FIGO system. Optimal cytoreduction was defined as the absence of macroscopic disease on the completion of the surgical procedure.

OS was defined as the length of time from diagnosis to death, or to the last follow-up examination of patients who are still alive. PFS was defined as the time interval from primary treatment where in the patient’s condition did not worsen. Clinical response of administration, including NAC, IDS, and adjuvant chemotherapy, was defined according to the standards of the Response Evaluation Criteria in Solid Tumors [26]. The pathology of all patients was initially reviewed by pathologists from JICR (Hou and Xu). A panel of pathologic markers was routinely measured. This study was approved by the ethics committee of the Jiangsu Institute of Cancer Research. Informed consent was obtained from all involved participants.

### Statistical analysis

The association between survival and adjuvant chemotherapy regime, courses, and therapy response was assessed by the Cox proportional hazards model. The multivariate model was constructed by step-wise regression techniques. *P*-values of less than 0.05 were considered to be significant. Survival distributions were estimated by the Kaplan-Meier method, and statistical significance was determined by log-rank test and Cox’s proportional hazard model analysis. Optimal IDS-related potential factors were explored by logistic regression analysis. All data manipulation and statistical analysis were performed by SPSS software v16 (SPSS for Windows, Rel.16. Chicago: SPSS Inc.).

## Results

### Patient characteristics

Among the 382 patients with advanced stage HG-SOC with large volume of ascites underwent entire primary therapy procedure in JICR, 160 (41.9%) patients received NAC and IDS, meeting the study criteria; 129 (80.1%) patients received subsequent optimal debulking surgery; and 31 patients received suboptimal debulking surgery (macroscopic residual disease). The median number of NAC cycles was 3 (range, 2 to 4). The clinicopathological characteristics of recruited cases are described in Table 1. The median follow-up duration of the survivors was 45 months (interquartile range, 38.2 to 56.4 months).

**Table 1:** Patient characteristics of the study population

Characteristic	Median	Percentage/range
Age (years)	62.2	36–82
Initial CA-125 level (U/mL)	920	41–24440
CA-125 decreasing kinetics	2.2	0.9–28.6
Tumor sites ^a^		
< = 3	38	23.7%
> 3	122	76.3%
Preoperative ascites		
< 500 ml	88	55.0%
> =500 ml	72	45.0%
Surgical residual		
No macroscopic focus	129	80.6%
1–2 cm	6	3.8%
> 2 cm	23	14.4%
Unknown	2	1.3%
FIGO stage ^b^		
IIIc	133	83.1%
IV	27	16.9%

Tumor sites ^a^ abdomen is divided into four quadrants according to the belly button

FIGO ^b^ the International Federation of Gynecology and Obstetrics

### Predictors of optimal IDS

The optimal IDS subset had a median preoperative serum CA-125 level of 445 U/mL (range, 45–730 U/mL) and CA-125 decreasing kinetics of 2.8. The suboptimal IDS group had a median preoperative serum CA-125 of 653 U/mL (range, 41–980 U/mL) and CA-125 decreasing kinetics of 1.7.

**Fig. 1:**
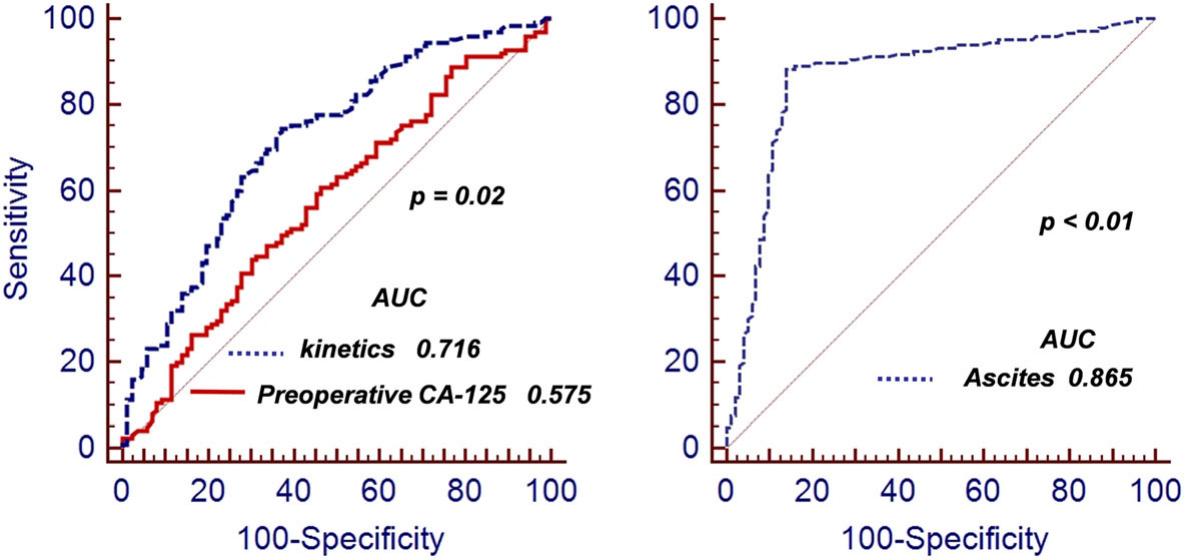
ROC curve for the outcome of interval cytoreduction surgery by preoperative serum CA-125 level, decreasing kinetics, and ascites volume

Univariate logistic regression analysis revealed that ascites regression, serum CA-125 level and decreasing kinetics, tumor sites, and patient’s age and status were IDS outcome-related factors (Table 2). Furthermore, we found that ascites regression (*p* = 0.01), preoperative serum CA-125 level (*p* = 0.02), and CA-125 decreasing kinetics (*p* = 0.01) were independent optimal IDS predictors in multivariate analyses (Table 2). Analysis with the receiver operating characteristic (ROC) curve revealed that CA-125 decreasing kinetics (*p* = 0.02) and preoperative ascites volume (*p* < 0.01) might imply optimal IDS (Fig. 1a and b).

**Table 2:** Logistic regression of optimal IDS associated factors in advanced HG-SOCs

Factors	Univariate		Multivariate	
	Exp(β)	Sig	Exp(β)	Sig
Age	1.01	0.15	1.00	0.52
Ascites	1.90	0.01	2.20	0.01
Stage	1.4	0.30	1.33	0.18
Tumor sites	1.3	0.22	1.16	0.47
Baseline CA-125	1.13	0.01	1.08	0.35
Preoperative CA-125	1.28	0.00	1.05	0.02
CA-125 decreasing kinetics	2.15	0.01	2.40	0.01

### Ascites regression was associated with prognosis

Using the univariate Cox proportional hazards model, we found that OS and PFS were associated with IDS outcome (*p* < 0.01 and *p* < 0.01, respectively), preoperative serum CA-125 level (*p* = 0.02 and *p* = 0.04, respectively), CA-125 decreasing kinetics (*p* < 0.01 and *p* < 0.01, respectively), and ascites volume (*p* < 0.01 and *p* < 0.01, respectively) as shown in Table 3. Multivariate analysis revealed that OS and PFS were independently associated with CA-125 decreasing kinetics (*p* = 0.04 and *p* < 0.01, respectively), IDS outcome (*p* < 0.01 and *p* < 0.01, respectively), and ascite volume (*p* = 0.03 and *p* = 0.02, respectively) as shown in Table 3.

**Table 3:** Survival-related characteristics in advanced HG-SOCs who underwent NAC/IDS

Variable	Univariate				Multivariate			
	PFS		OS		PFS		OS	
FIGO stage								
IV	1.00	(reference)	1.00	(reference)	1.00	(reference)	1.00	(reference)
IIIc	3.03	(0.35–13.66)	4.24	(0.52–6.95)	2.28	(0.30–4.10)	3.57	(0.48–6.46)
Ascites								
> =500 ml	1.00	(reference)	1.00	(reference)	1.00	(reference)	1.00	(reference)
< 500 ml	1.82	(1.45–2.85)	1.90	(1.56–2.98)	2.05	(1.40–2.95)	2.02	(1.66–4.08)
Outcome of surgery								
Suboptimal	1.00	(reference)	1.00	(reference)	1.00	(reference)	1.00	(reference)
Optimal	1.52	(1.15–2.42)	1.66	(1.22–2.50)	1.93	(1.75–2.84)	2.20	(1.40–4.24)
Tumor sites								
> 3	1.00	(reference)	1.00	(reference)	1.00	(reference)	1.00	(reference)
< = 3	1.32	(1.12–1.80)	1.49	(1.25–2.25)	1.26	(0.94–1.74)	1.40	(0.85–2.04)
Baseline CA-125	1.02	(1.01–1.08)	1.03	(1.01–1.08)	1.02	(0.97–1.07)	1.02	(0.98–1.06)
Preoperative CA-125	1.01	(1.00–1.04)	1.01	(1.00–1.04)	1.02	(0.96–1.05)	1.02	(0.96–1.05)
CA-125 decreasing kinetics	1.02	(1.00–1.04)	1.02	(1.00–1.04)	1.01	(1.00–1.04)	1.01	(1.00–1.05)

**Fig. 2:**
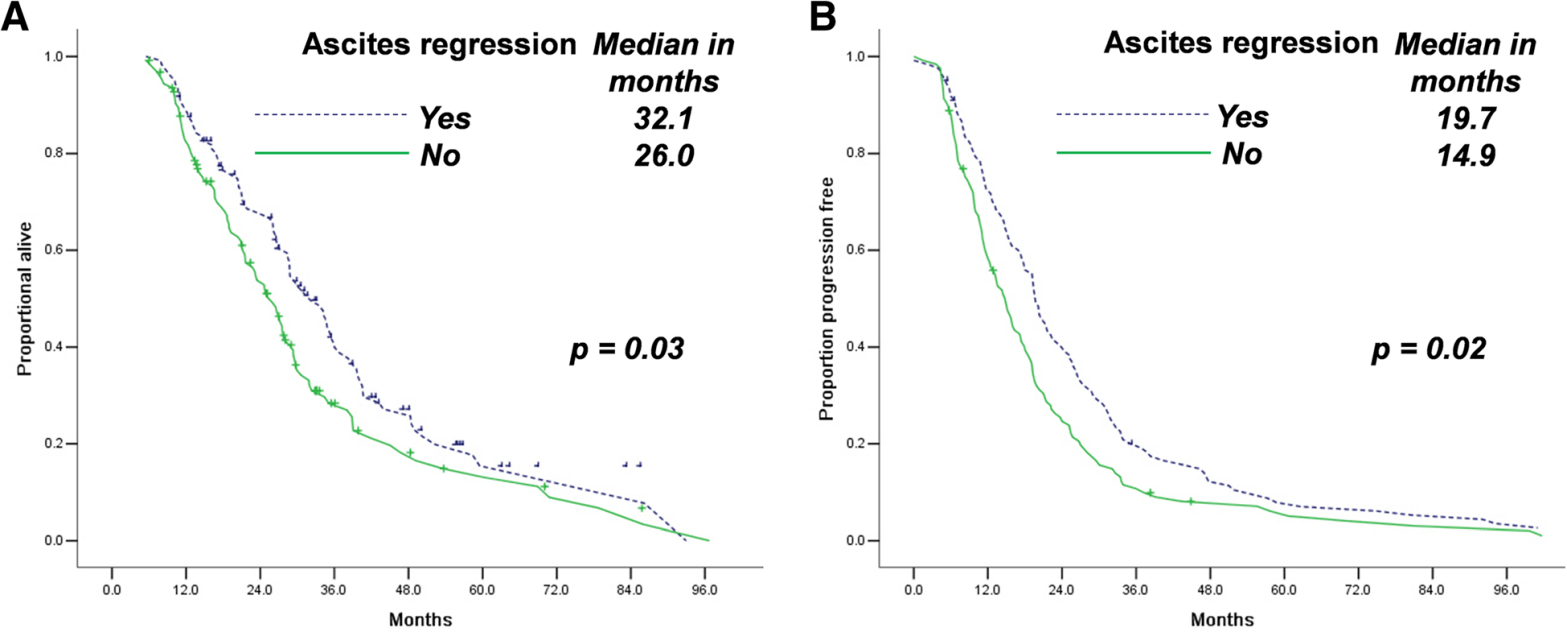
Ascites regression (**a**, **b**) is associated with longer OS and PFS in patients with advanced stage HG-SOC who underwent NAC/IDS

**Fig. 3:**
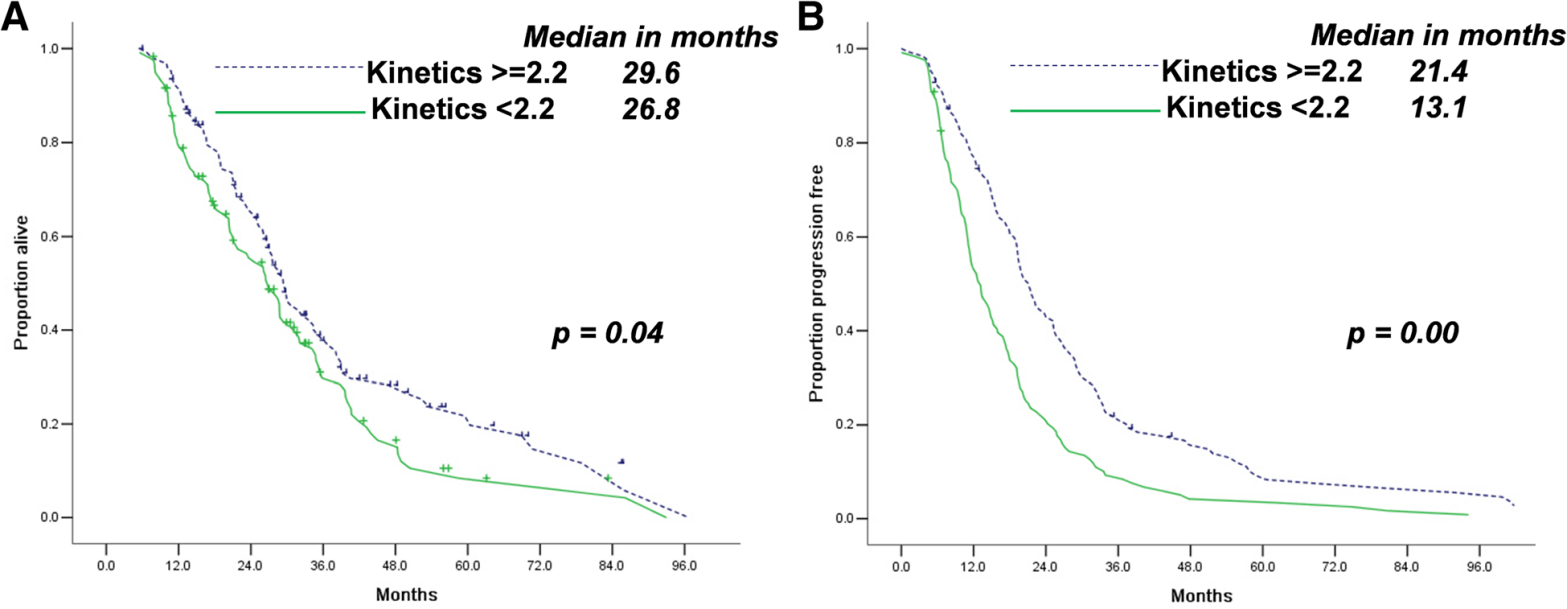
Higher CA-125 decreasing kinetics (**a**, **b**) is associated with longer OS and PFS in patients with advanced HG-SOCs who underwent NAC/IDS

Ascites regression indicated longer OS durations (32.1 months, 95% confidence interval [CI] 27.1–37.1 vs. 26.0 months, [CI] 22.3–29.6, Fig. 2a) and PFS (19.7 months, [CI] 17.8–21.6 vs. 14.9 months, [CI] 12.5–17.2, Fig. 2b) for patients who underwent IDS. We set the cut-off point at 2.2 for CA-125 decreasing kinetics. Groups with higher CA-125 decreasing kinetics had higher OS (29.6 months, [CI] 26.1–33.0 vs. 26.8 months, [CI] 22.7–30.1) and PFS (21.4 months, [CI] 18.6–24.2 vs. 13.1 months, [CI] 10.8–15.5) durations than groups with lower CA-125 decreasing kinetics (Fig. 3a and b).

## Discussion

Ovarian cancer is not a single disease entity, but rather comprises a heterogeneous group of tumors with distinct clinicopathological characteristics. Inconsistent outcomes regarding the prognostic significance of NAC may be related to the failure to consider tumor grade, histotype, stage, and chemotherapy response [27–32]. In the current study, we identified optimal IDS and prognosis-related factors in advanced HG-EOC patients who underwent NAC.

We suggested that patient stratification by ascites regression may be useful in analyzing the surgical timing of NAC in advanced HG-EOC. Based on our previous analysis [22–24], we assumed that CA-125 decreasing kinetics implied NAC response and followed IDS outcomes. Stratification by serum CA-125 level and CA-125 decreasing kinetics revealed that NAC group patients with preoperative serum CA-125 levels <450 U/ml and decreasing kinetics >2.2 significantly benefited in PFS and OS. This result implied that only selected patients, such as patients with HG-SOC and higher serum CA-125 levels and drastically reduced CA-125 kinetics, may receive survival benefit from NAC. Our data also suggested that biomarkers or other prediction models may be applied to identify target groups that can benefit from NAC. The most intriguing feature of the current data is the trend of the NAC hazard ratio according to the spectrum of preoperative ascites volume. Patients with a large amount of ascites had significantly poorer IDS outcomes compared with patients who exhibited ascites regression. This result suggests that NAC provides superior IDS outcome in a select subset of patients.

In the present cohort, optimal IDS rate was significantly higher in patients with CA-125 decreasing kinetics higher than 2.2 or ascites regression. Therefore, it was naturally expected that the improved optimal resection rate by NAC resulted in improved survival outcome. Reports had repeatedly stated that the improved optimal debulking rate obtained by NAC/IDS cannot directly translate into increased PFS or OS compared with PDS [33–36]. Although chemotherapy-assisted optimal cytoreduction is not biologically equivalent to optimal surgic al debulking, NAC also aims to increase the optimal resection rate. Our data proved that the patient subset exhibiting ascites regression had as uperior optimal resection rate. Therefore, ascites regression should be regarded as a timing indicator for IDS. Ascites regression may be associated with histotype, chemotherapy response, tumor volume and sites, and even invasion depth [37–42]. The higher optimal resection rate and chemotherapy response rate accompany by the disappearance of ascites may explain the association between ascites regression and prognosis.

Up to present, there were four randomized clinical trials comparing PDS and NAC followed by IDS for women with advanced ovarian cancer [36, 43–45]. These trials demonstrated that NAC/IDS was noninferior to PDS with respect to PFS and OS and resulted in a lower incidence of treatment-related morbidity and mortality. In EORTC 55971 [36], residual tumor of 1 cm or less was achieved in 42% of patients in the PDS arm and in 81% of patients in the NAC/IDS arm. Cytoreduction to < =1 cm residual disease was achieved in 41% of patients in the PDS arm and 73% of patients in the NAC/IDS arm (*p* = 0.0001) in the CHORUS trial [43]. The SCORPION [44, 46] trial found that complete cytoreduction was achieved in 58% of women in the NAC/IDS arm and 46% of women in the PDS arm, with a shorter median operative time in the NAC/IDS arm. In JCOG0602 [45], Optimal debulking proportions in NAC/IDS and PDS were 82 and 37%, respectively. Most of HG-SOCs are sensitive to platinum-based chemotherapy, and optimal debulking proportions increased in patients who underwent NAC. The rate of optimal cytoreduction in our study is up to 80.6%, similar to existing trials. It is very unlikely that an optimal surgical cytoreduction can be achieved in all HG-SOC patients who underwent NAC from literatures to the present study.

This retrospective analysis has several limitations. Firstly, unavoidable selection biases are inherent to its design. Given the long durations of our study, the influence of the administration change such as the emergence of new chemotherapy and molecular target agents, the improvement of disease evaluate strategy is difficult to exclude. The relatively severe recruited criteria may partly eliminate the influence of selected factors. Secondly, the absence of unified recruited standard for NAC/IDS and limited sample size also causes bias though MDT was set to indentify cases. Last but not least, recruited advanced stage HG-SOCs who underwent NAC/IDS were relatively older and comparatively frail. It cannot be translated to all EOCs directly until studies with accurate inclusion and exclusion criteria are available. Overall, given the selection bias of this non-blinded study, the application of these favorable outcomes to all epithelial ovarian carcinoma patients should be with caution.

## Conclusions

Our study supports the emerging evidence that ascites regression in advanced HG-SOCs is a predictor of optimal IDS. Ascites regression should be considered as an independent prognostic factor for both OS and PFS.

## Abbreviations

EOC: Epithelial ovarian cancer
IDS: Interval cytoreduction surgery
NAC: Neoadjuvant chemotherapy
OS: Overall survival
PDS: Primary debulking surgery
PFS: Progression free survival
